# Genetic Characterization of *Shigella flexneri* Isolates in Guizhou Province, China

**DOI:** 10.1371/journal.pone.0116708

**Published:** 2015-01-24

**Authors:** Shijun Li, Qiangzheng Sun, Xiaoyu Wei, John D. Klena, Jianping Wang, Ying Liu, Kecheng Tian, Xia Luo, Changyun Ye, Jianguo Xu, Dingming Wang, Guangpeng Tang

**Affiliations:** 1 Institute of Communicable Disease Control and Prevention, Guizhou Provincial Center for Disease Control and Prevention, 101 Bageyan Road, Guiyang, 550004, Guizhou, People’s Republic of China; 2 State Key Laboratory for Infectious Disease Prevention and Control, Collaborative Innovation Center for Diagnosis and Treatment of Infectious Diseases, National Institute for Communicable Disease Control and Prevention, China CDC, P.O. Box 5, Changping, Beijing, China; 3 International Emerging Infections Program, US Centers for Disease Control and Prevention, Beijing, China; 4 Global Disease Detection Branch, Division of Global Health Protection, Center for Global Health, Centers for Disease Control and Prevention, Atlanta, Georgia, United States of America; Cornell University, UNITED STATES

## Abstract

*Shigella flexneri* is one of the major etiologic causes of shigellosis in Guizhou Province, China. However, the genetic characteristics of circulating isolates are unknown. Phenotypic and molecular profiles of 60 *S. flexneri* isolates recovered in Guizhou between 1972 to 1982 and 2008 to 2010 were determined. Nine serotypes (1a, 2a, 3a, 1b, 2b, X, Y, 4av and Yv) were identified. Multi-locus sequence typing differentiated the isolates into 20 sequence types (STs); 18 were novel. Four STs, ST 129, ST 100, ST 126 and ST 18, were most abundant, accounting for 65% of the isolates. Thirty-nine *Not*I-pulsed field gel electrophoresis patterns (pulsotypes, PTs) were observed; eight PTs were represented by more than one isolate with six isolates sharing the PT 13 profile. Multi-locus variable-nucleotide tandem-repeat analysis recognized 44 different types (MTs); seven MTs were represented by more than one isolate and MT 1 was most commonly encountered. Correlation between genetic relationships and serotypes was observed among the isolates studied; the majority of isolates belonging to the same serotype from different years clustered together based on the molecular data. These clustered isolates were also from similar geographical origins. These results enhance our understanding of genetic relationships between *S. flexneri* in Guizhou Province and can be used to help understand the changing etiology of shigellosis in China.

## Introduction

Shigellosis, gastroenteritis caused by *Shigella* spp., is a major public health problem in both developing and developed countries [1, 2]. Approximately 164.7 million cases of shigellosis occur annually worldwide, resulting in 1.1 million deaths, primarily among children aged < 5 years [3]. Shigellosis occurs mainly in developing countries due to poor hygiene and limited access to clean drinking water; in industrialized countries the disease mainly results from travel to developing countries and exposure to contaminated foods and/or food handlers [1]. In China, *Shigella* spp. is one of the most frequently isolated gastrointestinal pathogens [4], accounting for up to 1.7 million episodes of shigellosis annually, with up to 200,000 patients admitted to hospitals [5, 6].

Guizhou Province, with nearly 50 million people, is an under-developed province in the southwest of China. Shigellosis has been one of the primary bacterial diseases in Guizhou in past decades, and from 2007 to 2010, 48,222 cases of shigellosis were reported [7]. Four species of *Shigella* can cause shigellosis but *Shigella flexneri* is the predominant species in China. Although there has been an observed shift in prevalence from *S*. *flexneri* to *Shigella sonnei* in recent years, *S*. *flexneri* is still one of the major etiologic cause of shigellosis in Guizhou Province [7].

Although *Shigella* has been a major source of disease over the past decades, information on the genetic relationships of circulating *S*. *flexneri* isolates from Guizhou Province is lacking. In this study, the molecular techniques multi-locus sequence typing (MLST), pulsed field gel electrophoresis (PFGE) and multi-locus variable-nucleotide tandem-repeat analysis (MLVA) were used to analyze the relationships between *S*. *flexneri* isolates recovered from Guizhou during the periods 1972 to 1982 and 2008 to 2010.

## Material and Methods

### Bacterial isolates and serotyping

Sixty isolates of *S*. *flexneri*, including 30 isolates recovered from 1972 to 1982 and 30 recovered from 2008 to 2010 in Guizhou Province, were analyzed (Table 1). The isolates were from Guiyang, Anshun, Qianxinan, Qiandongnan, Qiannan, Tongren and Zunyi Prefectures (seven of the nine prefectures making up Guizhou Province). All *S*. *flexneri* isolates were serotyped by slide agglutination using a commercially available monovalent antisera kit (Denka Seiken, Tokyo, Japan) and monoclonal antibody reagents (Reagensia AB, Sweden) per the manufacturer’s instructions [8]. *S*. *flexneri* isolates were routinly cultured in a 37°C incubator on Luria-Burtani (LB) agar plates or in an orbital shaker in LB broth.

**Table 1:** Isolation location, year and serotyping results of 60 *S*. *flexneri* isolates, Guizhou, 1972 to 1982 and 2008 to 2010.

No.	Isolate No.	Prefecture	County	Year	Serotype
F01	1972GZ01	Tongren	Songtao	1972	2a
F02	1972GZ02	Tongren	Songtao	1972	2a
F03	1973GZ01	Guiyang	Guiyang	1973	y
F04	1973GZ03	Guiyang	Guiyang	1973	4av
F05	1973GZ02	Guiyang	Guiyang	1973	2a
F06	1973GZ04	Guiyang	Guiyang	1973	3a
F07	1973GZ05	Guiyang	Guiyang	1973	3a
F08	1973GZ06	Guiyang	Guiyang	1973	3a
F09	1973GZ07	Guiyang	Guiyang	1973	3a
F10	1973GZ08	Guiyang	Guiyang	1973	2a
F11	1978GZ01	Qiandongnan	Kaili	1978	Yv
F12	1981GZ01	Zunyi	Qiannan	1981	1a
F13	1981GZ02	Zunyi	Qiannan	1981	1a
F14	1982GZ01	Qianxinan	Qinglong	1982	3a
F15	1982GZ02	Qianxinan	Puan	1982	1a
F16	1982GZ03	Qianxinan	Ceheng	1982	2a
F17	1982GZ04	Qianxinan	Zengfeng	1982	1a
F18	1982GZ05	Guiyang	Guiyang	1982	1a
F19	1982GZ06	Guiyang	Guiyang	1982	1a
F20	1982GZ07	Guiyang	Guiyang	1982	1a
F22	1982GZ09	Guiyang	Guiyang	1982	y
F23	1982GZ10	Zunyi	Zunyi	1982	1a
F24	1982GZ11	Guiyang	Guiyang	1982	1a
F25	1982GZ12	Guiyang	Guiyang	1982	1a
F26	1982GZ13	Zunyi	Zunyi	1982	1a
F27	1982GZ14	Zunyi	Zunyi	1982	1a
F28	1982GZ15	Zunyi	Zunyi	1982	1a
F29	1982GZ16	Zunyi	Zunyi	1982	1a
F30	1982GZ17	Zunyi	Zunyi	1982	1b
F31	1982GZ18	Zunyi	Zunyi	1982	1a
F32	2008GZ01	Guiyang	Kaiyang	2008	2a
F33	2008GZ02	Guiyang	Kaiyang	2008	2a
F34	2008GZ03	Anshun	Ziyun	2008	3a
F35	2008GZ07	Anshun	Pingba	2008	2a
F36	2008GZ08	Anshun	Pingba	2008	2a
F37	2008GZ11	Guiyang	Kaiyang	2008	3a
F38	2008GZ12	Guiyang	Kaiyang	2008	3a
F39	2008GZ13	Guiyang	Kaiyang	2008	3a
F40	2008GZ14	Guiyang	Kaiyang	2008	3a
F41	2008GZ15	Guiyang	Kaiyang	2008	3a
F42	2008GZ16	Guiyang	Kaiyang	2008	3a
F43	2008GZ17	Guiyang	Kaiyang	2008	3a
F44	2009GZ01	Guiyang	Kaiyang	2009	2a
F45	2009GZ02	Guiyang	Kaiyang	2009	2a
F46	2009GZ03	Anshun	Pingba	2009	2a
F47	2009GZ04	Anshun	Pingba	2009	2b
F48	2009GZ05	Anshun	Pingba	2009	2a
F49	2009GZ06	Anshun	Pingba	2009	2a
F50	2009GZ23	Anshun	Ziyun	2009	3a
F51	2009GZ25	Anshun	Ziyun	2009	2a
F52	2009GZ28	Guiyang	Kaiyang	2009	2a
F53	2009GZ29	Guiyang	Kaiyang	2009	2a
F54	2009GZ60	Guiyang	Kaiyang	2009	2a
F55	2009GZ68	Anshun	Pingba	2009	1a
F56	2010GZ01	Anshun	Ziyun	2010	2a
F57	2010GZ02	Anshun	Ziyun	2010	2a
F58	2010GZ03	Anshun	Ziyun	2010	x
F59	2010GZ04	Anshun	Ziyun	2010	x
F60	2010GZ05	Anshun	Ziyun	2010	2a
F61	2010GZ06	Anshun	Ziyun	2010	2a

### Preparation of DNA

Genomic DNA for PCR was prepared directly from bacterial colonies by the lysis by boiling method [8]. Briefly, a single colony from an overnight culture at 37°C on LB agar was suspended in 30 μl of distilled water and boiled at 100°C for 10 min. The sample was immediately cooled on ice for 5 min and centrifuged at 13,000 × *g* at 4°C for 10 min. The supernatant, containing DNA, was used as the template for PCR amplification.

### MLST

MLST analysis of 15 housekeeping genes was performed as described on the EcMLST website (http://www.shigatox.net/ecmlst). PCR products were sequenced bi-directionally.

Each unique allele was assigned a different number and the allelic profile (string of fifteen allelic loci) was used to define each isolate’s sequence type (ST). New allele numbers and STs were submitted to the EcMLST curator for confirmation and allocation of a unique identifier. Clustering and minimum spanning tree (MST) analysis was used to infer relationships among the isolates using the fingerprint analysis software BioNumerics version 4.5 (Applied Maths, Kortrijk, Belgium) [9].

### PFGE

PFGE analysis was performed using the method described by Ye *et al*. [9] using the restriction enzyme *Not*I. PFGE images were analyzed using BioNumerics. A PFGE pulsotype (PT) was defined as a pattern with one or more DNA bands different from other patterns. A dendrogram constructed using PFGE patterns was generated using the UPGMA algorithm with Dice-predicted similarity value of two patterns set at 1.0% pattern optimization and 0.8% band position tolerance.

### MLVA

MLVA was performed as previously described [10]. Eight VNTR loci (SF3, SF4, SF6, SF7, SF8, SF9, SF10 and SF25) were selected. The forward primer for each primer set was labeled at its 5′ end with a compatible HEX, FAM, TAMRA, and ROX dye, respectively. The loci were amplified individually, with each 20 μl PCR mixture containing 1 μl each primer, 1 μl DNA template, 10 μl 2 × Taq MasterMix (Cowin Biotech, Beijing, China) and deionized water used to make up volume differences to 20 μl. PCR products were analyzed by capillary electrophoresis on an ABI 3730XL sequencer with GeneScan 500 LIZ Size Standard (Applied Biosystems Incorporated, Carlsbad, CA, USA) as described [11]. The copy number of each VNTR locus was incorporated into BioNumerics software and analyzed as described previously [12]. Each unique allelic string was designated a unique MLVA type (MT). A dendrogram was constructed by UPGMA clustering based on categorical coefficient analysis.

## Results

### Distribution of serotypes

The 60 *S*. *flexneri* isolates were grouped into nine serotypes (1a, 2a, 1b, 2b, 3a, X, Y, 4av and Yv) (Table 1). Three serotypes, 1a, 2a and 3a, were predominant. Serotype 1a was the most frequently identified serotype (50%, 15/30) among isolates from 1972 to 1982, however 2a was dominated from 2008 to 2010 (56.7%, 17/30) of isolates. Further, 93.8% (15/16) of the 1a isolates were from 1972 to1982, and 72.3% (17/22) of the 2a isolates were isolated during 2008 to 2010; 3a isolates were almost equally distributed across both time periods. Isolate expressing serotype 4av (1973GZ03) and Yv (1978GZ01), recently described by Sun *et al* [13–15], were recovered from diarrheal cases in 1973 and 1978, respectively.

### MLST based genotypes

**Fig 1:**
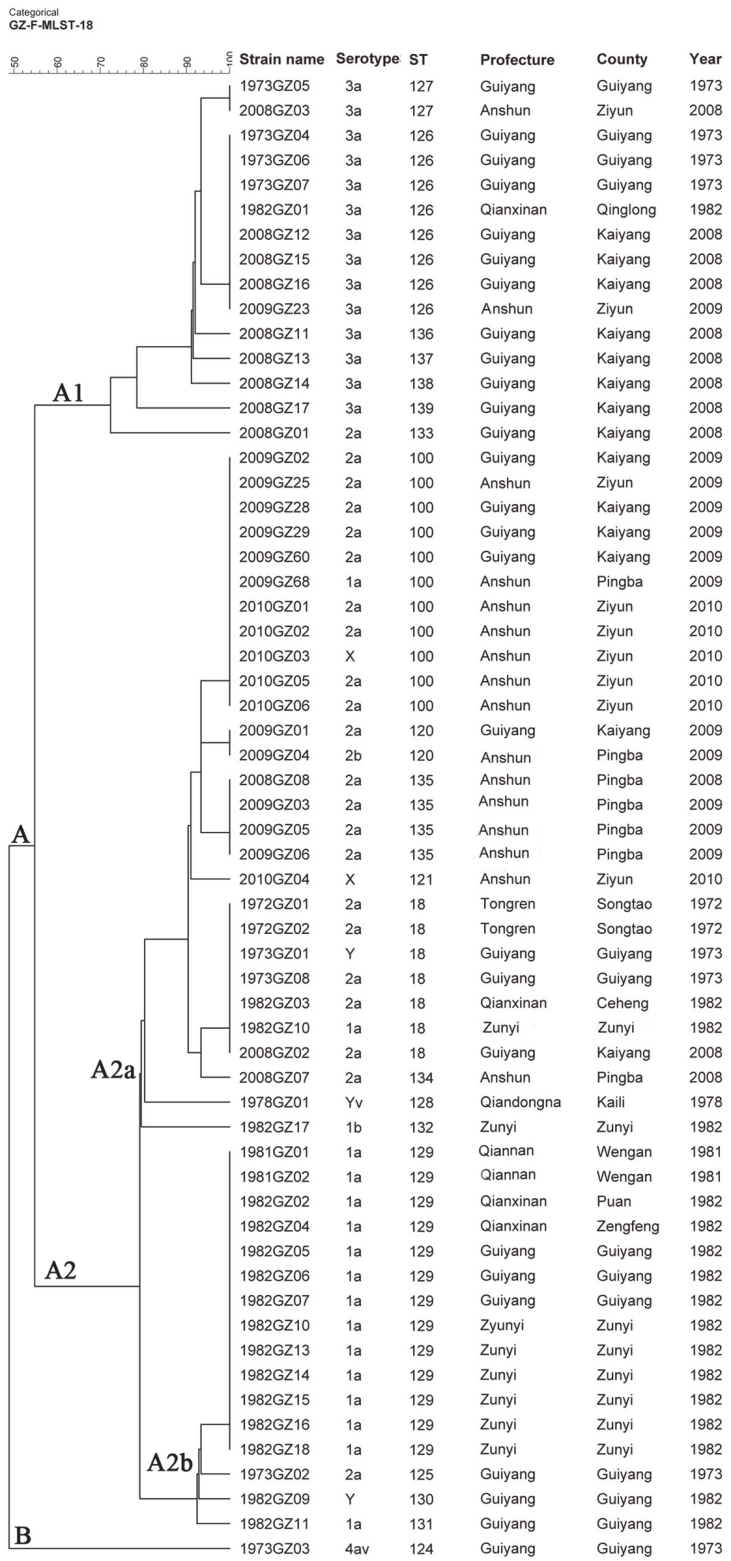
MLST clustering tree of *S*. *flexneri* isolates, Guizhou, from 1972 to 1982 and 2008 to 2010. The 60 isolates from Guizhou province were analyzed using a 15 allele MLST as described in the Materials and Methods.

**Fig 2:**
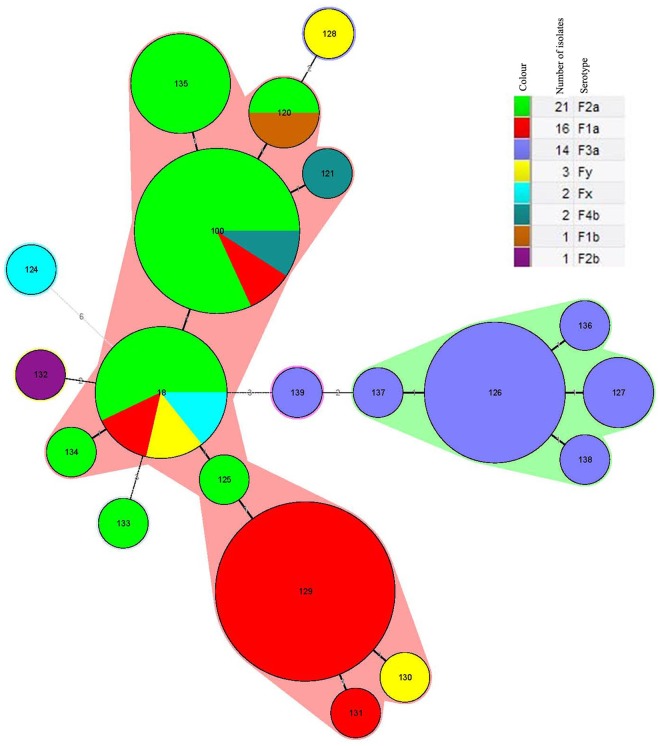
Genetic relationships of *S*. *flexneri* isolates recovered from Guizhou Province based on MLST. The minimum spanning tree was constructed using the 20 identified STs obtained from the 60 Guizhou Province isolates. Each circle corresponds to a single ST. The shadow zones in different color correspond to different clonal complexes. The size of the circle is proportional to the number of the isolates, and the color within the cycles represents the serotypes of the isolates. The corresponding color, serotype, number of isolates and back ground information are shown on the right of the minimum spanning tree.

The 60 isolates were divided into 20 STs, among which 2 STs (ST 18 and ST 100) have been previously reported; the remaining 18 STs (ST 120, ST 121, ST 124—ST 139) were unique (Table 2). The most common STs identified were ST 129 (22%), including isolates of serotype 1a; ST 100 (18%) including isolates of serotype 1a, 2a and X; ST 126 (13%) including all 3a isolates and ST 18 (12%) including isolates of serotype 1a, 2a, and Y. Among the most common STs, ST 18 (except for isolate 2008GZ02) and ST 129 isolates were recovered from 1972 to 1982; ST 100 isolates were only recovered from 2009 to 2010; and ST 126 isolates were recovered from both time periods, respectively. Eleven STs (18%) were singletons (Fig. 1). The predominant ST from 1972 to 1982 was ST 129 (43.3%, 13/30), while ST 100 was the predominant ST (36.7%, 11/30) from 2008 to 2010. A MLST cluster tree of the isolates showed they were divided into two clusters, designated A and B, with an overall coefficient of similarity of 50% (Fig. 1). ST 124 (4av, isolate 1973GZ03 isolated in 1973) was the only isolate within cluster B, while the remaining 19 STs formed cluster A. Cluster A was further divided in to subclusters A1 (15 isolates) and A2 (44 isolates); all the isolates in cluster A1 belonged to serotype 3a with the exception of one serotype 2a (2008GZ01) isolate. The STs in cluster A1 included ST 126, ST 127, ST 133 and ST 136–139. Cluster A2 was further divided into two distinct branches A2a (28 isolates) and A2b (16 isolates); A2a included 20 of the 22 *S*. *flexneri* 2a isolates, 2 1a, 2 X, and one each of 1b, 2b, Y and Yv. Branch A2b contained 14 of the 16 *S*. *flexneri* 1a isolates and a single 2a and Y isolate. The cluster tree indicated that isolates belonging to the same serotype closely clustered based on the time of isolation. A minimum spanning tree (MST), based on the 20 STs indicated that 20 STs were divided into 2 clonal complexes (CCs) (CC 18 and CC 126) and four singletons; CC18 contains isolates expressing serotypes 1a, 2a, 2b, 4b, X and Y, and included 10 STs. In contrast all isolates in CC 126 were serotype 3a and included five STs; ST 124, ST 132, ST 133 and ST 139 were singletons (Fig. 2).

**Table 2:** *S*. *flexneri* MLST allelic profiles and ST designation, Guizhou, 1972 to 1982 and 2008 to 2010.

IsolatesName	Alle profile															ST
	arcA	aroE	aspC	clpX	cyaA	dNaG	fadD	grpE	icdA	lysP	mdh	mtlD	mutS	rpoS	uidA	
1972GZ01	8	10	13	16	10	11	14	10	16	11	19	15	14	15	14	18
1972GZ02	8	10	13	16	10	11	14	10	16	11	19	15	14	15	14	18
1973GZ01	8	10	13	16	10	11	14	10	16	11	19	15	14	15	14	18
1973GZ02	8	33	159	16	33	11	14	10	16	11	179	15	37	15	236	124
1973GZ03	8	10	13	16	10	11	230	10	16	11	19	15	14	15	14	125
1973GZ04	8	35	15	16	10	11	14	30	30	11	193	15	14	73	14	126
1973GZ05	8	35	15	16	10	11	14	30	207	11	193	15	14	73	14	127
1973GZ06	8	35	15	16	10	11	14	30	30	11	193	15	14	73	14	126
1973GZ07	8	35	15	16	10	11	14	30	30	11	193	15	14	73	14	126
1973GZ08	8	10	13	16	10	11	14	10	16	11	19	15	14	15	14	18
1978GZ-01	8	10	15	16	10	11	229	10	16	127	19	15	14	15	14	128
1981GZ01	28	10	13	16	10	11	230	10	16	11	19	15	14	15	14	129
1981GZ02	28	10	13	16	10	11	230	10	16	11	19	15	14	15	14	129
1982GZ01	8	35	15	16	10	11	14	30	30	11	193	15	14	73	14	126
1982GZ02	28	10	13	16	10	11	230	10	16	11	19	15	14	15	14	129
1982GZ03	8	10	13	16	10	11	14	10	16	11	19	15	14	15	14	18
1982GZ04	28	10	13	16	10	11	230	10	16	11	19	15	14	15	14	129
1982GZ05	28	10	13	16	10	11	230	10	16	11	19	15	14	15	14	129
1982GZ06	28	10	13	16	10	11	230	10	16	11	19	15	14	15	14	129
1982GZ07	28	10	13	16	10	11	230	10	16	11	19	15	14	15	14	129
1982GZ09	28	10	13	189	10	11	230	10	16	11	19	15	14	15	14	130
1982GZ10	28	10	13	16	10	11	230	10	16	11	19	15	14	15	14	129
1982GZ11	28	10	13	16	10	11	230	10	16	11	19	15	14	72	14	131
1982GZ12	8	10	13	16	10	11	14	10	16	11	19	15	14	15	14	18
1982GZ13	28	10	13	16	10	11	230	10	16	11	19	15	14	15	14	129
1982GZ14	28	10	13	16	10	11	230	10	16	11	19	15	14	15	14	129
1982GZ15	28	10	13	16	10	11	230	10	16	11	19	15	14	15	14	129
1982GZ16	28	10	13	16	10	11	230	10	16	11	19	15	14	15	14	129
1982GZ17	8	34	13	189	10	11	14	10	16	11	19	15	14	15	14	132
1982GZ18	28	10	13	16	10	11	230	10	16	11	19	15	14	15	14	129
2008GZ01	8	10	15	16	10	11	14	10	207	11	19	15	14	73	14	133
2008GZ02	8	10	13	16	10	11	14	10	16	11	19	15	14	15	14	18
2008GZ03	8	35	15	16	10	11	14	30	207	11	193	15	14	73	14	127
2008GZ07	8	10	171	16	10	11	14	10	16	11	19	15	14	15	14	134
2008GZ08	8	10	171	16	10	11	14	10	16	23	19	15	14	15	14	135
2008GZ11	8	35	15	16	10	11	14	30	30	23	193	15	14	73	14	136
2008GZ12	8	35	15	16	10	11	14	30	30	11	193	15	14	73	14	126
2008GZ13	8	35	13	16	10	11	14	30	30	11	193	15	14	73	14	137
2008GZ14	8	35	15	16	10	11	14	30	30	11	193	15	17	73	14	138
2008GZ15	8	35	15	16	10	11	14	30	30	11	193	15	14	73	14	126
2008GZ16	8	35	15	16	10	11	14	30	30	11	193	15	14	73	14	126
2008GZ17	8	35	13	16	10	11	14	30	30	11	19	15	14	15	14	139
2009GZ01	8	10	15	16	10	11	14	10	16	23	19	15	14	15	14	120
2009GZ02	8	10	13	16	10	11	14	10	16	23	19	15	14	15	14	100
2009GZ03	8	10	171	16	10	11	14	10	16	23	19	15	14	15	14	135
2009GZ04	8	10	15	16	10	11	14	10	16	23	19	15	14	15	14	120
2009GZ05	8	10	171	16	10	11	14	10	16	23	19	15	14	15	14	135
2009GZ06	8	10	171	16	10	11	14	10	16	23	19	15	14	15	14	135
2009GZ23	8	35	15	16	10	11	14	30	30	11	193	15	14	73	14	126
2009GZ25	8	10	13	16	10	11	14	10	16	23	19	15	14	15	14	100
2009GZ28	8	10	13	16	10	11	14	10	16	23	19	15	14	15	14	100
2009GZ29	8	10	13	16	10	11	14	10	16	23	19	15	14	15	14	100
2009GZ60	8	10	13	16	10	11	14	10	16	23	19	15	14	15	14	100
2009GZ68	8	10	13	16	10	11	14	10	16	23	19	15	14	15	14	100
2010GZ01	8	10	13	16	10	11	14	10	16	23	19	15	14	15	14	100
2010GZ02	8	10	13	16	10	11	14	10	16	23	19	15	14	15	14	100
2010GZ03	8	10	13	16	10	11	14	10	16	23	19	15	14	15	14	100
2010GZ04	8	10	13	16	10	11	14	10	16	23	19	15	14	17	14	121
2010GZ05	8	10	13	16	10	11	14	10	16	23	19	15	14	15	14	100
2010GZ06	8	10	13	16	10	11	14	10	16	23	19	15	14	15	14	100

### PFGE based Genotypes

**Fig 3:**
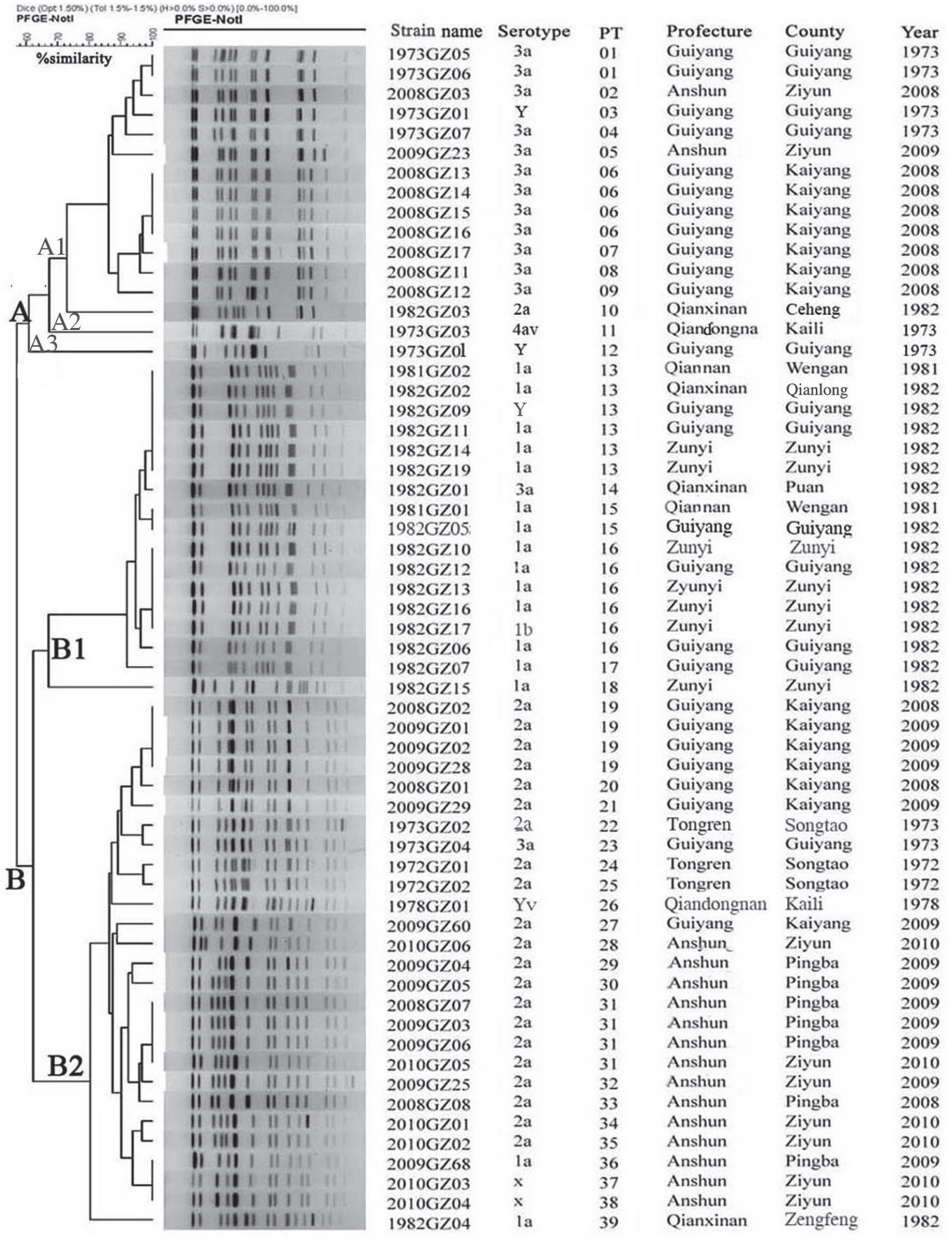
Relationship of *S*. *flexneri* isolates recovered from Guizhou based on *Not*I-PFGE analysis. The dendrogram were constructed using UPGMA. The corresponding PFGE pattern, serotype and background information are shown to the right of the dendrogram.

The genotypes and genetic relatedness of the Guizhou *S*. *flexneri* isolates were also determined using PFGE. *NotI*-digested *S*. *flexneri* DNA generated 39 reproducible unique PTs, each with 12–17 bands. Eight patterns were represented by more than one isolate with PT 13 containing the greatest number of isolates, followed by PT 16. Among the isolates from 1972 to 1982, the predominant PT was PT 13 or PT 16, each representing 20.0% (6/30) of the total, while the predominant PT among isolates from 2008 to 2010 was PT 06, PT 19 or PT 31, each representing 13.3% (4/30) of the total. All 60 isolates were related at a coefficient of similarity of 60%, but two main clusters could be distinguished at a 62% similarity value (cluster A and B; Fig. 3). Cluster A was split into two additional broad subgroups, A1 (15 isolates) and A2 (1 isolate). The majority (12 of 14) serotype 3a isolates, with the exception of isolates 1973GZ04 and 1982GZ01, grouped together in A1; single isolates of serotype 2a, 4av and Y complete this group. Subgroup A2 contained a single Y serotype isolate. Cluster B split into two subgroups as well, subgroup B1 (17 isolates) and B2 (27 isolates). The majority of serotype 1a isolates (14 of 16) were found in subgroup B1; the three remaining isolates expressed 1b, 3a and Y. Subgroup 2B contained 21 of the 22 serotype 2a isolates, 2 isolates of 1a and X and single isolates of 3a and Yv. Similar to MLST, the majority of isolates expressing the same serotype were closely clustered and this was also related to the year of isolation. Additionally, the isolate (isolate 1973GZ03 recovered in 1973) of serotype 4av, was formed cluster A3, while the serotype Yv isolate, shared a unique but similar PT and was included in subgroup B2.

### MLVA typing

**Fig 4:**
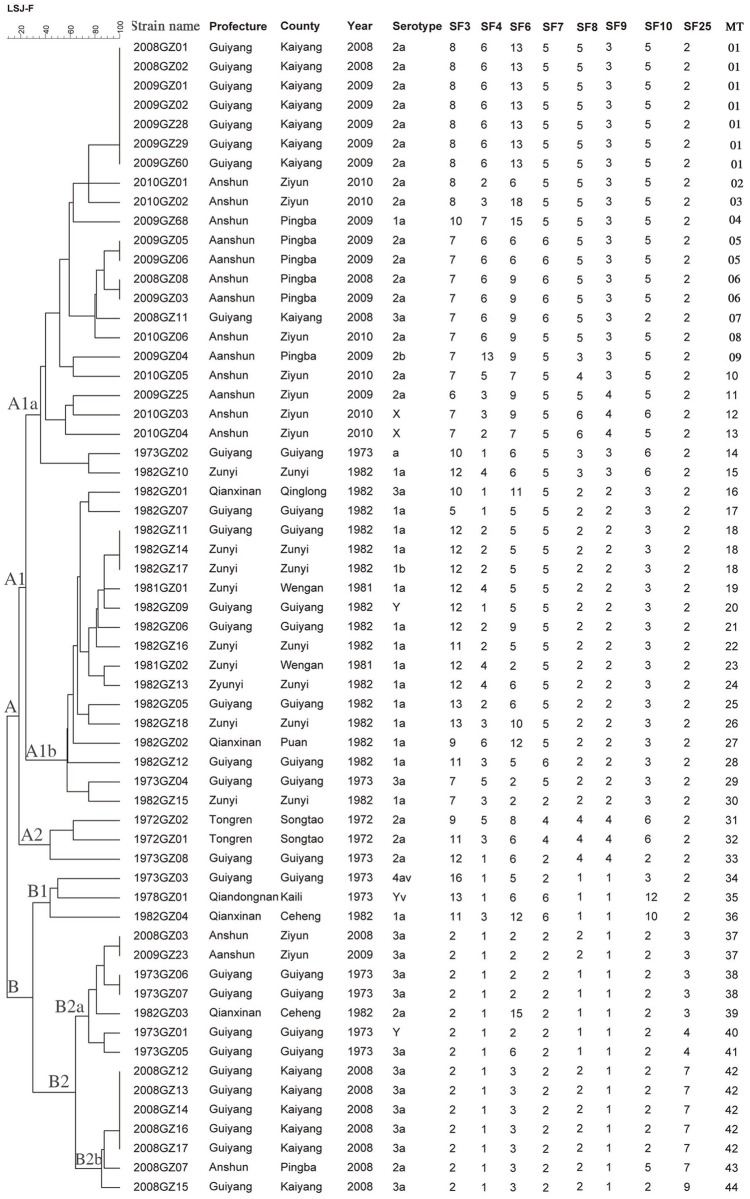
Relationship of *S*. *flexneri* isolates recovered from Guizhou based on MLVA. Isolates were analyzed using an eight VNTR loci MLVA scheme. The dendrogram was constructed using UPGMA. The corresponding MLVA type with copy numbers for the eight VNTRs, serotype, and background information are shown to the right of the dendrogram.

Using MLVA, the 60 *S*. *flexneri* isolates grouped to 44 different MTs (Fig. 4). Seven MTs were represented by more than one isolate with MT 1 occurring most frequently (n = 7) followed by MT 42 (n = 5). The predominant MT among isolates recovered from 2008 to 2010 was MT 1 (23.3%; 7/30), however the predominant MT among isolates from 1972 to 1982 was not obvious. MLVA analysis showed the greatest diversity among the 60 *S*. *flexneri* isolates resulting in an extensively branched tree. Like MLST and PFGE, two main clusters, A and B, were observed (Fig. 4). The majority of serotype 1a and 2a isolates were assigned in cluster A, in which the 1a and 2a isolates were further grouped in cluster A1 and A2, respectively, whereas most of the serotype 3a isolates were grouped in cluster B. Isolates of serotype X assigned to cluster A showed relatively close relationship to serotype 2a, while the serotype Y isolates were closely related to serotype 1a or 3a isolates. Isolates belonging to the same serotype but recovered from different years showed clear relatedness, indicated by grouping in the same clusters. For example, serotype 3a isolates recovered from 1972 to 1982 and 2008 to 2010 clustered together, and similar characteristics were also observed for isolates belonging to serotype 1a and 2a (Fig. 4). Serotype 4av and Yv isolates were closely grouped in cluster B1. Additionally, isolates belonging to the same serotype and within a close time span clustered together based on geographical origin. For example, 2a isolates recovered in Guiyang, Ziyun and Pingba shared very similar patterns.

## Discussion

In the present study, the genetic characteristics of 60 isolates of *S*. *flexneri* recovered from Guizhou Province between 1972 to 1982 and 2008 to 2010 were systematically studied. Thirty of the *S*. *flexneri* isolates were from shigellosis cases from 1972 to 1982 and the remaining isolates were recovered from patients with shigellosis from 2008 to 2010. The serotypes of the isolates used in this study included 1a, 2a, 3a 1b, 2b, X, Y, 4av and Yv; isolates were also from seven of the nine prefectures in Guizhou Province. Serotyping results indicated that *S*. *flexneri* 1a and 2a were the predominant serotypes from 1972 to 1982 (94%) and 2008 to 2010 (72%), respectively, while 3a isolates were almost equally recovered in both periods. The predominant serotype (2a) in Guizhou Province recovered from 2008 to 2010 are consistent with isolates from Suzhou of Jiangsu, Henan and Shanxi Province [16–18], but are different from isolates of other provinces such as Beijing (4a and 4b) and Jiading of Shanghai Province [19, 20]. It is noteworthy that isolates of serotype 4av and Yv were recovered as early as 1973 and 1978, respectively, indicating the early emergence of these serotypes in China.

Recently a number of genotyping methods with higher discriminatory power than serotyping or biochemical testing such as MLST [21, 22], PFGE [5, 23] and MLVA [10, 11, 24] were introduced to characterize *Shigella* isolates. These methods are based primarily based on changes in isolate genotype, permitting analysis of phylogenetic relationships. Analysis of the isolates can be helpful for clinical diagnosis, treatment, prevention and control of shigellosis. Choi *et al*. [22] showed that *S*. *flexneri* serotypes 1–5, X and Y clustered together in a group containing many allelic variants while serotype 6 formed a distinct group, as previously established [25, 26]. Wang *et al*.[10] reported that phylogenetic groupings of 242 *S*. *flexneri* isolates recovered from shigellosis cases in Taiwan between 1995 to 2008, based on PFGE and MLVA profiles, correlated with serotype and isolate origin. Two distinct clusters for isolates of serotype 3 were shown but only one distinct cluster for each of the serotype groups 1a/1b/NT, 2a/2b/X/NT, 4a/Y, and 6 were revealed. Serologically different isolates including serotype Y and subserotype 4a; serotype X and subserotype 2b; subserotypes 1a and 1b, and subserotypes 3a and 3b, were genetically more closely related than indicated by serotyping alone.

Ye *et al*. [9] previously analyzed 37 serotype X and 69 serotype 1a, 2a, 2b, 3a, 4a, 5b, and Y isolates from China; all belonged to ST91 (later renamed ST 100), and concluded that *S*. *flexneri* epidemics in China have been caused by a single epidemic clone, ST 100. In this study, 60 isolates of *S*. *flexneri* from Guizhou Province separated into 20 STs based on a 15 loci MLST scheme; 18 of the STs were novel. The most common STs from 1972 to 1982 were ST 18, ST 126 and ST 129, however, ST 100 and ST 126 appeared between 2008 to 2010. Our results suggested that the predominant ST was ST 129 from 1972 to 1982, while ST 100 was the the predominant ST during 2008 to 2010, and the predominant ST is consistent with ST of isolates from other provinces of China [9]. Isolates belonging to the same serotype clustered in accordance with the year of isolation using all three genotyping approaches. MST indicated that the 20 STs were divided into 2 CCs, CC 18 and CC 126, and 4 singletons. CC 18 contained isolates expressing serotypes 1a, 2a, 2b, 4b, X and Y, while all the isolates in CC 126 belonged to serotype 3a. In addition, both the cluster tree and the MST, based on MLST data, showed that the isolates of serotype 4av (1973GZ03) was distant from the isolates belonging to other serotypes.

PFGE is a broadly applicable typing method with a high degree of intra- and inter laboratory reproducibility when standardized protocols are followed [23]. It has been shown to be a powerful tool in the laboratory for discriminating *Shigella* isolates during an outbreak [27]. In this study, PFGE discriminated the 60 isolates of *S*. *flexneri* into 39 unique PFGE patterns. Isolates belonging to the same serotypes mainly clustered together; the most closely related isolates were temporally associated with one another was well, suggesting that some drift was associated within each serotype over time. For instance, isolates of serotype 2a in cluster B2 isolated during the period of 1972 to 1982 and 2008 to 2010 were closely clustered, respectively, and similar clustering characteristics were observed for the isolates belonging to serotype 1a (cluster B1) and 3a (cluster A1), but some isolates, such as 2008GZ03, 1982GZ03 and 2009GZ23, were assigned irrespective their isolation time. Additionally, isolates with similar geographic origin were also often grouped by PFGE together as they tended to express the same serotypes.

MLVA is a prominent typing tool which has been used for characterizing *S*. *flexneri*; it has also been a useful tool for phylogenetic analysis [10]. In the present study, the *S*. *flexneri* isolates were discriminated into 44 different MTs and showed a low (approximately 20%) coefficient of similarity, indicating the high discriminatory power of the MLVA method. This finding is consistent with a previous study showing MLVA exhibited a discriminatory power greater than PFGE [10]. For most of isolates belonging to serotypes 1a, 2a and 3a, MLVA results correlated with serotyping. However, isolates of serotype X and Y were associated with serotype 1a and 2a isolates, respectively, and the serotype 4av isolate was closely related to serotype 3a isolates; this is similar to results observed previously [10]. Similar to PFGE, the majority of isolates belonging to the same serotype were temporally and geographically related.

In this study, isolates 1973GZ03 and 1978GZ01were serologically identified as serotype 4av and Yv respectively. These serotypes differ from 4a and Y because they react with monoclonal antibody MASF IV-1 [13–15]. In theory, serotype 4av and Yv should have originated from serotype 4a and Y, primarily differing only in the acquisition of a 6.8k plasmid carrying a phospheantransferase gene (*opt*) responding for the MASF IV-1 antigenic determinant in these isolates [13–15]. In this study, isolates of serotype of 4av (1973GZ03) and Yv (1978GZ01) were typed as ST 124 and ST 128 with MLST, respectively. MLST showed that 4av isolate were genetically distinct from isolates belonging to other serotypes, while Yv isolates were relatively close to isolates of serotype 2a. Molecular analysis indicates that isolate 1978GZ01 carries a dysfunctional *gtrII* gene within genome, and hence it is genetically similar to isolates from serotype 2a (unpublished data). This would explain the observed similarity between isolate 1978GZ01 and the majority of serotype 2a isolates.

## Conclusions

In conclusion, phenotypic and molecular profiles of 60 *S*. *flexneri* isolates recovered in Guizhou between 1972 to 1982 and 2008 to 2010 were analysed. Nine serotypes (1a, 2a, 3a, 1b, 2b, X, Y, 4av and Yv) were identified, and the predominant serotype has changed from 1a to 2a in Guizhou Province. MLST differentiated the isolates into 20 sequence types (STs); 18 were novel. Four STs, ST 129, ST 100, ST 126 and ST 18, were most abundant, accounting for 65% of the isolates. The predominant ST was ST 129 in 1972 to 1982, while ST 100 was the predominant ST during 2008 to 2010. Thirty-nine *Not*I-PFGE (pulsotypes, PTs) were observed; eight PTs were represented by more than one isolate with six isolates sharing the PT 13 profile. MLVA analysis recognized 44 different types (MTs); seven MTs were represented by more than one isolate and MT 1 was most commonly encountered. Correlation between genetic relationships and serotypes was observed among the isolates studied; the majority of isolates belonging to the same serotype from different years clustered together based on the molecular data. These clustered isolates were also from similar geographical origins.
